# A Combination of Nicotinamide and D-Ribose (RiaGev) Is Safe and Effective to Increase NAD^+^ Metabolome in Healthy Middle-Aged Adults: A Randomized, Triple-Blind, Placebo-Controlled, Cross-Over Pilot Clinical Trial

**DOI:** 10.3390/nu14112219

**Published:** 2022-05-26

**Authors:** Yongquan Xue, Trisha Shamp, G. A. Nagana Gowda, Michael Crabtree, Debasis Bagchi, Daniel Raftery

**Affiliations:** 1 Bioenergy Life Science, Inc., Ham Lake, MN 55304, USA; michael.crabtree@bioenergyls.com; 2 Prism Clinical Research Institute, Saint Paul, MN 55114, USA; t.shamp@nucleusnetwork.com; 3 Northwest Metabolomics Research Center, University of Washington, Seattle, WA 98109, USA; ngowda@uw.edu (G.A.N.G.); draftery@uw.edu (D.R.); 4 College of Pharmacy and Health Sciences, Texas Southern University, Houston, TX 77004, USA; dbagchi@adelphi.edu

**Keywords:** nicotinamide adenine dinucleotide (NAD^+^), aging, oxidative stress, cortisol, D-ribose, vitamin B3, energy homeostasis

## Abstract

Nicotinamide adenine dinucleotide (NAD^+^) is an essential cofactor required for proper functioning of all cells and its decline is correlated with advancing age and disease. This randomized, triple-blind, placebo-controlled, crossover pilot study assessed the efficacy and safety of a combination of nicotinamide with D-ribose (RiaGev) for NAD metabolome enhancement and related benefits in healthy middle-aged adults. Supplementing with 1520 mg RiaGev twice daily for 7 days significantly increased the NAD^+^ metabolome in blood, especially NADP^+^ by 27% compared to the placebo group (*p* = 0.033) and over the baseline (*p* = 0.007). Increases in glutathione and high energy phosphates were also observed in the blood. Seven-day supplementation with RiaGev significantly (*p* = 0.013) reduced overall blood glucose without significant changes in insulin secretion (*p* = 0.796), suggesting an improved insulin sensitivity and glucose tolerance. The waking salivary cortisol of the subjects steadily and significantly decreased (*p* = 0.026) in the RiaGev group in contrast to the placebo. Subjects in the RiaGev group showed less fatigue, improved mental concentration and motivation over the baseline (*p* = 0.015, 0.018, and 0.012, respectively) as observed through the Checklist Individual Strength (CIS) questionnaire. There were no clinically relevant adverse events, or alterations in hematology, electrolytes, liver, and kidney markers pre- and post-supplementation. RiaGev appears to be safe and efficacious in increasing NAD^+^ metabolome in healthy middle-aged adults, as shown by this study.

## 1. Introduction

The NAD^+^ metabolome, including NAD^+^, NADH, NADP^+^, and NADPH, are pivotal for human health. First, these are reusable coenzymes for diverse redox reactions and energy homeostasis. Second, NAD^+^ and NADP^+^ are consumable substrates in enzymatic reactions regulating crucial biochemical processes, including gene expression, energy homeostasis, DNA repair and regeneration, apoptotic cell death and lifespan, calcium signaling, glucose homeostasis, and circadian rhythms [1,2,3,4,5]. As coenzymes, the NAD^+^ metabolome participates in over 60% of reactions in cellular metabolism, and their homeostasis is the determining factor for maintaining redox balance and metabolism [1,2]. As a consumable substrate, the NAD^+^ concentration is directly linked with advancing age and premature aging [6] and fat composition [7]. NAD-consuming enzymes, including PARPs, sirtuins, and CD38, have far reaching implications in human health and disease [8], especially for aging and age-related chronic degenerative diseases.

There are four NAD^+^ biosynthetic pathways operating in mammals [9], including the *de novo* pathway starting from amino acid tryptophan, and three alternative routes of pyridine salvage. Theses pyridines, nicotinic acid (NA), nicotinamide (NAM), and nicotinamide riboside (NR), are collectively referred to as niacin or vitamin B3 [10]. They may arise from dietary intake and/or intracellular NAD^+^ catabolism. The starting material for the *de novo* pathway, tryptophan, is also obtained from dietary protein sources such as eggs, meat, and cheese. *De novo* NAD^+^ synthesis is generally considered to be insufficient to sustain normal NAD^+^ homeostasis [11].

Vitamin B3, commonly found in enriched food and beverages, is also limited in amount because nicotinic acid (NA) causes flushing when its dose is high enough [12]. Most NAD^+^ in mammals is synthesized from nicotinamide (NAM) via the amidated salvage route. NAM salvage is catalyzed by nicotinamide phosphoribosyl-transferase (NAMPT) [13], which regulates circadian rhythm [4]. Some researchers believe that age-related NAD^+^ decline is due to NAMPT decline with advancing age [14].

However, additional evidence to enhance the consumption of NAD^+^ by NAD^+^ consuming enzymes, such as PARPs, sirtuins, and CD38 has been reported [15]. More importantly, the age-related NAM salvaging capacity in human skeletal muscles can be reversed by exercise [16]. Therefore, it is possible to increase NAD^+^ by supplementing NAM on a regular basis.

The last decade has witnessed a significant volume of research on enhancing NAD^+^ by supplementing with nicotinamide riboside (NR) [17]. NR is a more distant precursor in the NAD^+^ biosynthetic pathway. It is believed that NR is converted into NMN by NR kinase (NRK1/2) using ATP as a co-substrate [18]. It was demonstrated that supplementation of NR increases NAD^+^ levels, enhances oxidative metabolism, and reduces fat and hepatic steatosis [17,18]. However, there are some controversies regarding how NR works to raise NAD^+^. Multiple experiments demonstrated that NR degraded very quickly into nicotinamide and ribose, especially when ingested orally [19,20]. Therefore, the reported benefits of NR could be attributable to circulating nicotinamide or nicotinamide and ribose. To address this issue, we initiated research into combinations of nicotinamide and D-ribose.

NAM is the preferred treatment for pellagra [21]. It is also used to treat acne and non-melanoma skin cancer [22]. More recently, NAM is considered to be a potential candidate to increase the NAD^+^ metabolome for anti-aging applications [23]. High-dose NAM indeed enhanced NAD^+^ levels and ameliorated disease in a rat model of obesity [24]. When considering its long-term application, we came across several limiting factors. The daily recommended dietary intake to prevent vitamin B3 deficiency is only approximately 15 mg in adults [25]. However, doses more than 3 g per day can also cause side effects including hepatotoxicity [26]. The daily tolerable upper intake of NAM is specified accordingly at 900 mg in the European Union (Opinion of the Scientific Committee on Food on the Tolerable Upper Intake Levels of Nicotinic Acid and Nicotinamide, issued by the Scientific Committee on Food), 500 mg in Canada, and 5 mg/kg in Japan (Overview of Dietary Reference Intakes for the Japanese, issued by Ministry of Health, Labor and Welfare). Therefore, it is only practical to use NAM within a relatively low dose range, such as 100–500 mg per day. It is important to mention that NAM is ineffective for boosting NAD+ at doses below 90 mg per day [27]. Beyond 900 mg, it poses a regulatory challenge. Therefore, maximizing its NAD^+^ boosting capability in a low dose range is preferable. Through a series of preclinical animal studies (results yet to be published), we developed a novel combination of NAM and D-ribose that amplifies the NAD-boosting capability of NAM and reduces its potential side effects. Here we report its first clinical trial in healthy volunteers.

This randomized, triple-blind, placebo-controlled, cross-over pilot study investigated the efficacy and safety of RiaGev via evaluation of the NAD^+^ metabolome and diverse health related parameters in healthy adults (age: 35–65 Y). We selected this age group because many health problems that directly affect healthy aging occur during this period. Additionally, this is the period of life that bears the heaviest burden of stress. Oxidative stress is a known factor for many chronic degenerative diseases and is detrimental to healthy aging [27,28]. Two of the most common chronic diseases that are accompanied by aging are obesity and diabetes. In 2016, the WHO reported that approximately 1.6 million deaths were due to diabetes, and half of these individuals had high blood glucose before the age of 70 [27]. Hence, it is crucial to actively control blood glucose and oxidative stress during the midlife period. Therefore, stress parameters and blood glucose were chosen as secondary outcomes following the primary outcome of modulating the NAD^+^ metabolome.

## 2. Materials and Methods

### 2.1. Clinical Trial Design and Subject Population

#### 2.1.1. Clinical Trial Design

**Figure 1:**
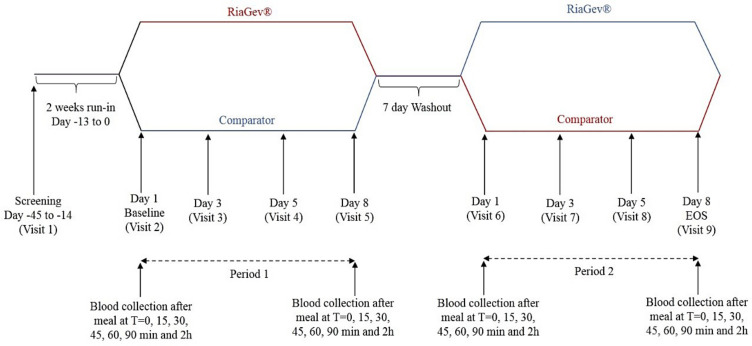
Clinical Trial Design During this randomized, triple-blind, placebo-controlled, crossover study, either RiaGev or Placebo (Comparator) was administered from Day 1 through Day 8. On Day 1, the treatment was provided after Baseline sample collection. On Day 8, the treatment was provided 2 h before sample collection. During the rest of Periods 1 or 2, treatment was administered twice daily, once before breakfast and once before dinner. Samples were collected on Day 1, Day 3, Day 5, and Day 8 for cofactors analysis. Oral glucose tolerance tests were conducted on Day 1 before treatments and on Day 8 after the treatments.

The clinical trial performed was a randomized, triple-blinded, placebo-controlled, cross-over study (Figure 1). After the first visit (Visit 1), the subjects were divided into two matching groups, one for RiaGev and the other for Placebo, to proceed through Period 1. When Period 1 was completed after eight days, the subjects were without treatment during a 7-day washout time and then crossed to the opposite treatment in Period 2. In each treating period, subjects made four visits to take measurements and sampling on Day 1, Day 3, Day 5, and Day 8. During Day 1, baseline measurements were made and RiaGev and Placebo treatments were distributed. On Day 3, Day 5, and Day 8, we measured the progression of the treatment. To minimize circadian effects, the visits and samplings were kept at approximately the same time of day on each visit.

#### 2.1.2. Subject Population

Subjects in this study were healthy, active, males and females between the ages of 35 and 65 years. The major inclusion criteria included: BMI between 18.5 and 29.9 kg/m^2^; female participants were not pregnant as indicated by a test; healthy as determined by laboratory results, medical history, physical exam and EKG; agreed to avoid supplementation with tryptophan and vitamin B3 or its derivatives (niacin, nicotinic acid, niacinamide) one week prior to randomization and during the study; had the ability to complete maximal and submaximal exercise tests; agreed to maintain current diet, activity level, and sleeping cycle throughout the study; agreed to comply to all study procedures with voluntary, written, informed consent to participate in the study.

The study excluded subjects with any known diseases or inflammatory conditions. Detailed inclusion and exclusion criteria are listed on ClinicalTrials.gov.

### 2.2. Investigational Product and Its Administration

#### 2.2.1. Investigational Product and Placebo

The investigational product (IP), RiaGev, contained 1280 mg of D-ribose, 240 mg of nicotinamide, and 480 mg of palm oil, packed into three capsules. D-ribose and nicotinamide are active ingredients and palm oil is the excipient. The batch number for RiaGev was S0776313.

The Placebo, contained 1280 mg dextrose and 480 mg palm oil, also packed in three capsules of same size and color as IP. Its batch number for the placebo was S1126314.

Both RiaGev and Placebo were provided by Bioenergy Life Science, Inc. (Ham Lake, MN, USA).

#### 2.2.2. Investigational Product Administration

Participants were instructed to take two doses of RiaGev or placebo daily, once in the morning and once in the evening, administered immediately before breakfast and dinner. In both study periods, on Day 1 only the evening dose was administered, and on Day 8 only the morning dose was administered.

### 2.3. Sample Collection

#### 2.3.1. Blood

Blood samples for NMR analysis was collected as follows: (1) Heparin plasma tubes were used for whole blood collection (BD vacutainer, sodium heparin 95 USP Units, REF 367878). (2) Blood was drawn, gently mixed by inverting the tube 5–6 times, and quickly made into 400 μL (accurately measured) aliquots of blood using 2.0 mL pre-cooled Eppendorf tubes at 4 °C on ice. (3) Immediately, the aliquots were frozen using a dry ice bucket and then transferred to a −80 °C freezer. (4) Frozen aliquots were shipped on dry ice to the Northwest Metabolomics Research Center at UW for analysis of the coenzymes. Enough dry ice was packed in the shipping box to ensure samples remained frozen until received.

#### 2.3.2. Saliva Sample Collection

Participants collected salivary cortisol samples using the Salivette collection device. Saliva samples were collected within 15 min of waking and prior to eating on the mornings of all visits, except screening (Visit 1). To ensure proper collection, participants were provided with instructions from the Prism clinical site.

### 2.4. Clinical Measurements and Additional Assessments

#### 2.4.1. Laboratory Methodologies

The safety endpoints of complete blood count (WBC count with differential, RBC count, hemoglobin, hematocrit, platelet count, RBC indices (MCV, MCH, MCHC, RDW)), liver function (AST, ALT, bilirubin), and kidney function laboratory blood tests (creatinine, eGFR, electrolytes) were analyzed from the blood drawn at visits 1 (screening), 5, 6, and 9 by HCMC Laboratory (Minneapolis, MN, USA), using standardized procedures. At visits 2, 5, 6, and 9 glucose and insulin were analyzed by HCMC Laboratory using standardized procedures.

From the blood samples obtained during visits 2–9, NAD^+^, NADP^+^, and NADPH were analyzed by Northwest Metabolomics Research Center, UW, Seattle using an established NMR methodology [29,30]. Glutathione (GSH), Glutathione disulfide (GSSG), adenosine triphosphate (ATP) and adenosine monophosphate (AMP) were analyzed by the Northwest Metabolomics Research Center from the blood samples provided during visits 2, 5, 6, and 9 by the same methodology [29].

Urine pregnancy tests (Henry Schein One Step+) were conducted at Prism Clinical for participants of childbearing capacity at visits 1 and 2.

#### 2.4.2. Checklist Individual Strength Questionnaire (CIS) Questionnaire

The Checklist Individual Strength Questionnaire is a validated questionnaire for healthy working people. This is a 20-item questionnaire scored on a 7-point scale measuring subjective fatigue experience, reduced concentration, reduced motivation, and reduced physical activity level [31]. The Questionnaire was administered to participants on visits 2 to 8.

#### 2.4.3. Food Records

Participants were asked to record their food consumption during the study. These data were used to calculate and analyze their daily calories, macronutrient and micronutrient intake throughout the study using Nutritics software (Nutritics, Dublin, Ireland, 2019). The food records were reviewed by trained staff at the visits, and participants were counseled with dietary suggestions as required.

#### 2.4.4. Study Compliance

Compliance to study procedures was recorded in the relevant section of the compliance report form (CRF) at each visit. Product compliance was assessed by counting the returned unused product at each visit. Compliance was calculated by determining the number of dosage units taken divided by the number of dosage units expected to have been taken multiplied by 100.

#### 2.4.5. Adverse Event (AEs) Monitoring

During the study, participants recorded AEs in their diary. At each visit, the participant was asked “have you experienced any difficulties or problems since I last saw you?” after which the answers (AEs) were documented in the study record and were classified as per the description, duration, intensity, frequency, and outcome. The PI assessed any AEs and decided causality.

### 2.5. Statistical Analyses

#### 2.5.1. Efficacy Analysis

Categorical variables, counts, and percentages were presented. The denominator for each percentage was the number of subjects within the study group unless otherwise specified. Possible differences between groups were assessed using two-tailed Chi-square or Fisher’s exact test, as appropriate.

For summaries of interval variables, the arithmetic means, standard deviations, medians, and minimum-maximum ranges were presented to two decimal places. Possible differences between groups at screening/baseline visits were assessed by ANOVA. For each group, the change in each outcome between study time points was assessed using a paired Student’s *t*-test if normally distributed or Wilcoxon Signed-Rank test if otherwise.

Changes in the primary outcome and each secondary outcome were compared between groups using repeated measures mixed model analysis of covariance (ANCOVA) if normally or log-normally distributed. Each model included the study group time (study visit) as a fixed effect, the baseline value of the dependent variable as a covariate, and subject as random effect. Between-group *p*-values were obtained from the final model.

#### 2.5.2. Safety Analysis

A descriptive analysis was provided for pre-emergent and post-emergent adverse events (AEs) reported in this study. Furthermore, the outcome and relationship to study products was reported for each AE that was classified as possibly or probably related to the study products. The number of participants with at least one AE was compared between study arms using Fisher’s Exact test.

Vital signs, hematology, and clinical chemistry parameters were summarized as means, standard deviations, medians, and minimum-maximum ranges. Changes from screening/baseline were assessed using the paired *t*-test.

#### 2.5.3. Level of Significance and Statistical Software

All hypothesis testing was carried out at the 5% (2-sided) significance level unless otherwise specified. *p*-values less than 0.01 were reported as <0.01 and those less than or equal to 0.05 were considered statistically significant. All analyses were performed using the R Statistical Package version 3.6.3 (R Core Team, Vienna, Austria, 2020) for Microsoft Windows.

## 3. Results

### 3.1. Subject Enrollment and Baseline Characteristics

This randomized, triple-blind, placebo-controlled, crossover pilot study, was conducted from October 2019 to November 2020. The protocol was approved by IntegReview IRB (protocol code 19RNHB (1918)) and the study is registered on ClinicalTrails.gov under identifier NCT04483011 [32].

Fifty healthy men and women between the ages of 36 and 65 years were screened for this study. Eighteen individuals who met the inclusion and exclusion criteria were recruited. These subjects were healthy as determined by laboratory results, medical history, physical examination, and EKG. Of these, nine subjects were randomly assigned to each group to proceed with either the RiaGev-Placebo or Placebo-RiaGev sequence. There were no significant differences in demographics or baseline clinical characteristics between the sequences (Table 1). During the study one subject was withdrawn from the RivGev-Placebo sequence after the first period. Therefore, the Intent to treat (ITT) population was 18 and per protocol (PP) population was 17. Statistical analyses were performed for both populations. Since their results are very much alike, only the result for the ITT population is presented. The compliance for taking RiaGev and Placebo supplementations is 100%.

### 3.2. Primary Outcome-NAD^+^ Metabolome

**Figure 2:**
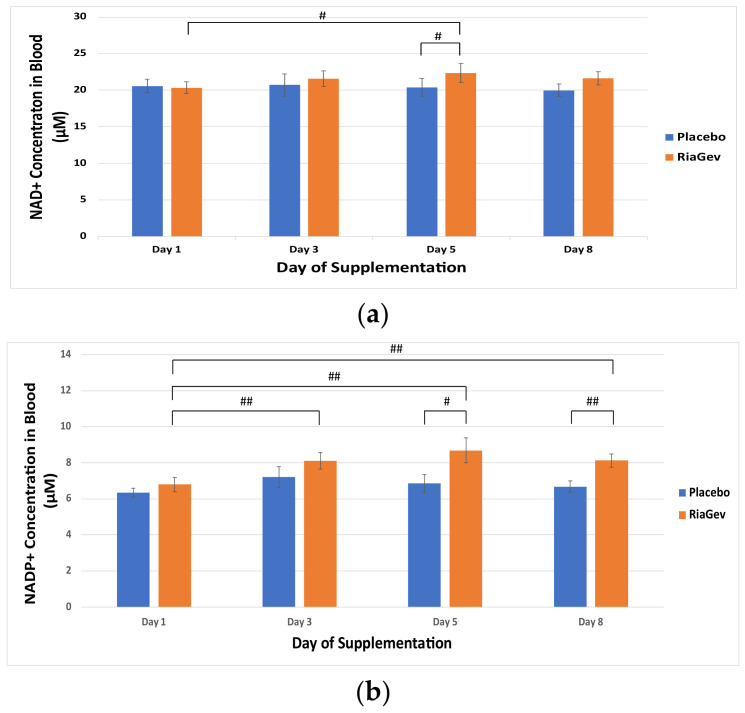

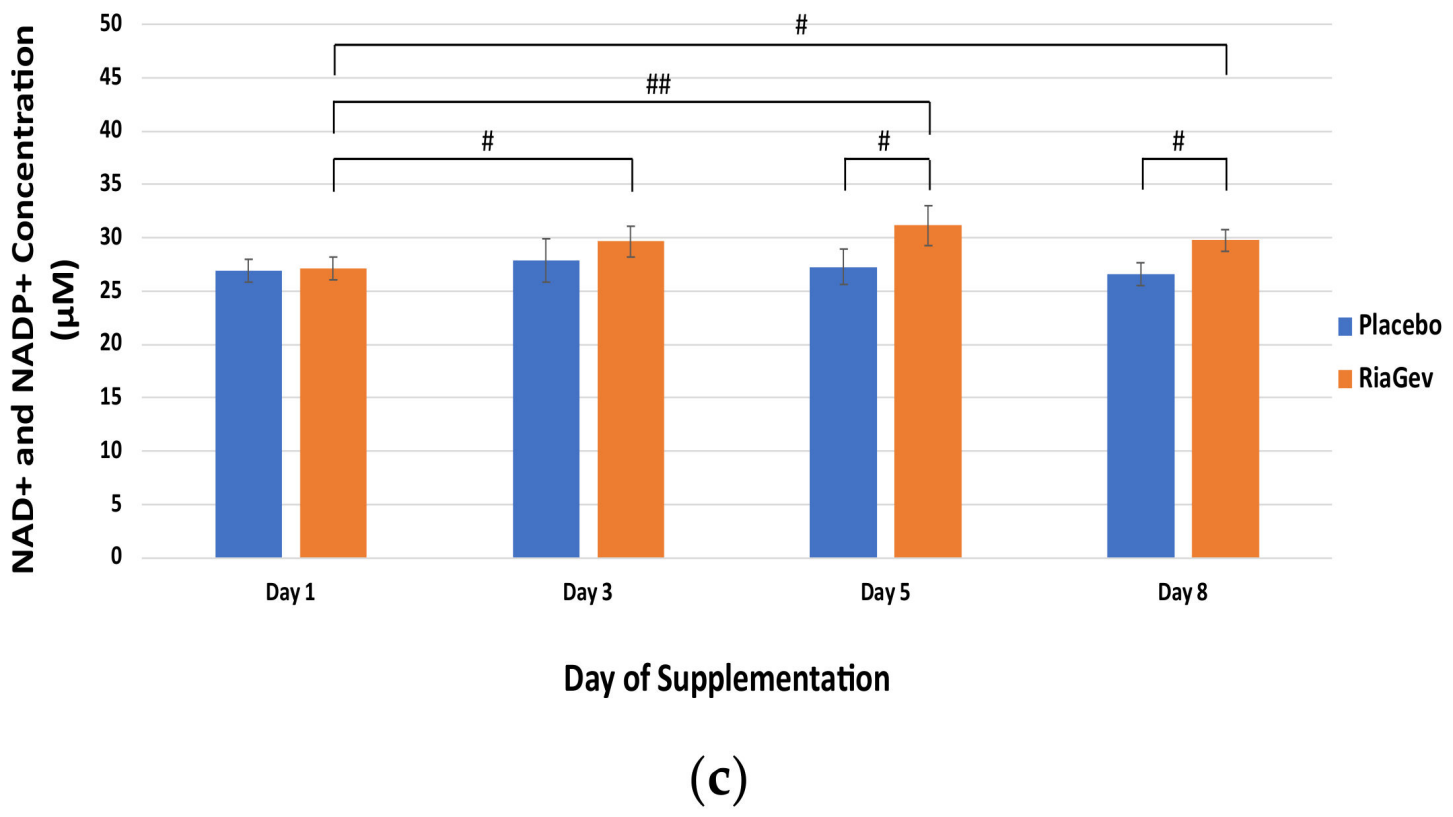
Whole Blood NAD^+^ and NADP^+^ Concentrations on Days 1, 3, 5, and 8 of the Clinical Trial. NAD^+^ and NADP^+^ levels are represented by solid bars with standard errors indicated. (**a**) NAD^+^ concentration. (**b**) NADP^+^ concentration. (**c**) Combined NAD^+^ and NADP^+^ concentration. Significant changes within the RiaGev group over time or significant differences between RiaGev and Placebo groups on the same day are marked by # (*p* < 0.05) or ## (*p* < 0.01).

The primary outcome of this study is the level of the NAD^+^ metabolome, especially the NAD^+^ concentration, in the blood after supplementing with RiaGev vs. Placebo. As shown in Figure 2a, the NAD^+^ concentration in the RiaGev group increased steadily over baseline after supplementation. At day 5, the NAD^+^ concentration in RiaGev group was significantly higher than that at Day 1, a 10.4% increase (*p* = 0.034), which is also significantly higher than that in the placebo group (*p* = 0.044). At Day 8, there is also a trending significant increase of 6.4% in NAD^+^ concentration over baseline (*p* = 0.07). By comparison, the blood NAD^+^ concentration in the placebo group did not change significantly over the period.

Compared with the mild increase in NAD^+^ level, an unexpectedly dramatic increase in NADP^+^ concentration was observed (Figure 2b). Significant within-group increases of 19.1%, 27.6%, and 19.6% were recorded with RiaGev group at Days 3, 5, and 8 over baseline, respectively (*p* ≤ 0.008). The NADP^+^ concentrations for the RiaGev group are also significantly greater than that of Placebo group at Day 5 and 8 (*p* ≤ 0.04).

NAD^+^ and NADP^+^ are the two NAD^+^ metabolome cofactors that increased the most by RiaGev supplementation in this pilot trial. When combined, NAD^+^ + NADP^+^ concentrations observed at Days 5 and 8 were significantly higher than that in Placebo (*p* ≤ 0.029, Figure 2c), in conjunction with significant within-group concentration increases of 9.4%, 14.8% and 9.7% reported within RiaGev group at Days 3, 5, and 8 respectively (*p* ≤ 0.032).

There was only a trending increase in NADPH in the RiaGev group compared to the Placebo group (*p* >0.08). However, when NADP^+^ and NADPH are considered together, the nicotinamide adenine dinucleotide phosphates in the RiaGev group increased significantly at 8.3%, 22.5%, and 12.9% at Days 3, 5, and 8, respectively, compared to the Placebo group and over the baseline (*p* < 0.05).

The NADH level was not measured in this study because the method [30] used to preserve NADPH during blood storage and shipment destroyed the NADH signal (please see the Supplementary Materials). Whole blood 1-methyl-nicotinamide (MeNAM) and nicotinic acid adenine dinucleotide phosphate (NAAD(P)) were below the NMR detection limit in the blood samples (Supplementary Materials).

### 3.3. Secondary Outcomes

#### 3.3.1. Blood Glucose and Insulin Concentration in OGTT

**Figure 3:**
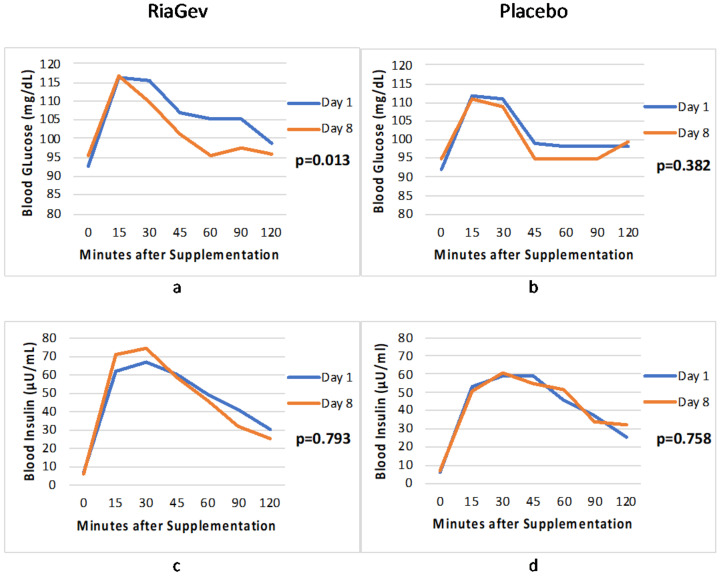
Oral Glucose Tolerance Test (OGTT) Profile Before (Day 1) and After Treatments (Day 8). (**a**) blood glucose in RiaGev group; (**b**) blood glucose in Placebo group; (**c**) blood insulin in RiaGev group; and (**d**) blood insulin in Placebo group. The *p*-value on each panel indicates its iAUC difference between Day 8 and Day 1. The blood glucose iAUC of the RiaGev group reduced significantly from Day 1 to Day 8 (*p* = 0.013) while its overall insulin secretion did not change significantly (*p* = 0.793). There is no significant change within the Placebo group for either blood glucose (*p* = 0.382) or insulin (*p* = 0.758).

The post-prandial oral glucose tolerance test (OGTT) was performed to determine the blood glucose and insulin response to a standardized meal before and after 7-day supplementation with RiaGev or Placebo. Figure 3a,c shows the blood glucose and insulin levels, respectively, of RiaGeb group post-prandial at Day 1 (before supplementation) vs. Day 8 (after seven days of supplementation). In Figure 3a, the blood glucose iAUC level of RiaGev group at Day 8 was significantly lower compared to Day 1 (1700.42 vs. 662.50, respectively, *p* = 0.013). However, the overall insulin level (Figure 3c) of RiaGev group at Day 8 was not significantly different from Day 1 (*p* = 0.793). When their peak values considered, the insulin peak on Day 8 was higher than on Day 1 (74 vs. 67 μU/mL, respectively, Figure 3c), despite the glucose peak remaining the same (116 vs. 114 mg/dL at 15 min post-prandial at Day 8 vs. Day 1, respectively, Figure 3a). As a result, the blood glucose concentration at Day 8 dropped more rapidly than at Day 1 (Figure 3a), suggesting an improved insulin sensitivity after 7-day RiaGev supplementation. On the contrary, either blood glucose or insulin profile (overall and peak) of the placebo group was not significantly different on Day 8 vs. Day 1 (Figure 3b,d).

When total blood glucose AUC (tAUC) is considered, it can be calculated as the total Area Under the Curve between 0 and 120 min (Figure 3a,b). RiaGev reduced blood glucose AUC 4.92% (from 12,771.15 on Day 1 to 12,143.17 on Day 8) after 7-day supplementation, while the Placebo group did not change significantly (12,041.55 on Day 1 vs. 11,914.8 on Day 8). It is important to note that the subjects’ blood glucose levels remain in the healthy range throughout the trial. The RiaGev group had a relatively larger, albeit insignificantly, blood glucose AUC on Day 1 to start with than the Placebo group did. It is unlikely that this insignificant baseline imbalance plays a role in the blood glucose reduction observed in this trial. Nevertheless, its implication will be discussed in the discussion section.

#### 3.3.2. Total Glutathione

**Figure 4:**
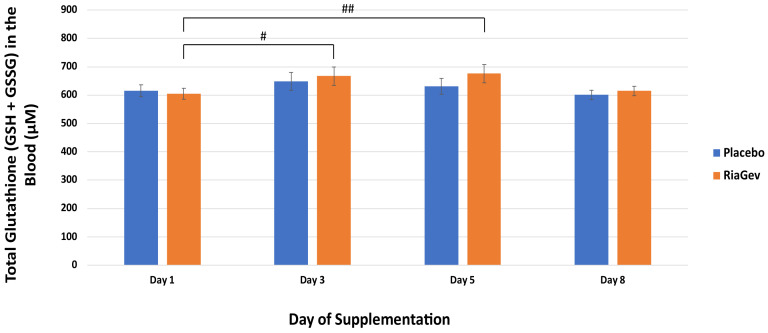
Whole Blood Glutathione (GSH and GSSG) Concentration on Days 1, 3, 5, and 8 of Clinical Trail. The total blood glutathione concentrations are represented as solid bars with standard errors indicated. Significant changes are marked by # (*p* < 0.05) or ## (*p* < 0.01).

Glutathione is a circulating antioxidant generated in the body. There was a significant increase of total glutathione (GSH + GSSG) at Day 3 and 5 (10.2% and 11.6%, respectively) in the RiaGev group over baseline (*p* ≤ 0.016), meanwhile the glutathione in the Placebo group had no significant change during the study period (Figure 4).

#### 3.3.3. High Energy Phosphates ATP + ADP

**Figure 5:**
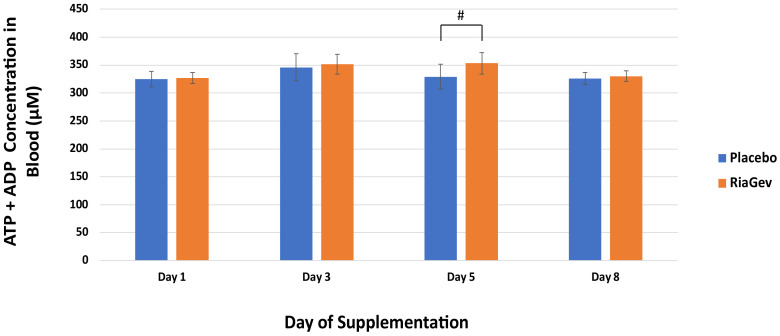
Whole blood ATP and ADP measurements on Days 1, 3, 5, and 8. ATP + ADP concentrations are represented by solid bars with standard errors indicated. Significant difference between the group is marked by # (*p* < 0.05).

In the clinical trial, RiaGev consistently increased the ATP/AMP ratio at Days 3, 5, and 8, and the ratio was also consistently higher in the RiaGev than in the Placebo group, although not significantly (data not shown). However, when the high energy phosphates are taken together, the RiaGev group consistently had more ATP + ADP than in the Placebo group at Days 3, 5, and 8 (Figure 5). This difference is significant on Day 5, when the RiaGev group was 7.3% higher than the Placebo group (*p* = 0.029).

#### 3.3.4. Salivary Cortisol

**Figure 6:**
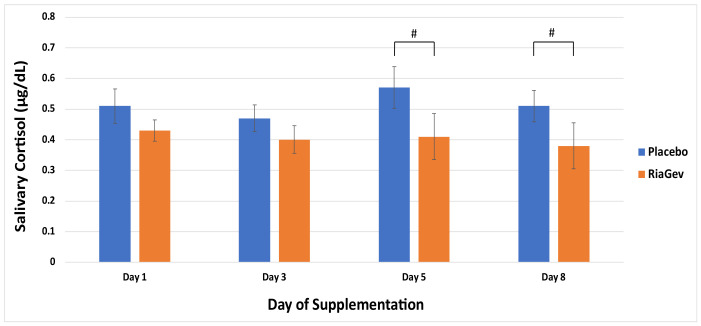
Waking Salivary Cortisol Measurements on Days 1, 3, 5, and 8. Salivary cortisol levels are represented by solid bars with standard errors indicated. A steady and significant decline of waking salivary cortisol from Day 1 baseline levels was observed during the 7-day RiaGev supplementation (orange bars), which contrasted with the higher and fluctuating cortisol levels in the placebo group (blue bars). Significant differences between the groups are marked by # (*p* < 0.05).

The waking salivary cortisol is presented in Figure 6. There was a significant between-group difference in waking salivary cortisol at Day 5 and Day 8, where the RiaGev group displayed lower levels of cortisol than the placebo group (*p* = 0.044). In the RiaGev group, the cortisol level steadily declined after Day 1 as supplementation continued, while its level fluctuated in the Placebo group at Days 3, 5, and 8.

#### 3.3.5. CIS Questionnaire

**Figure 7:**
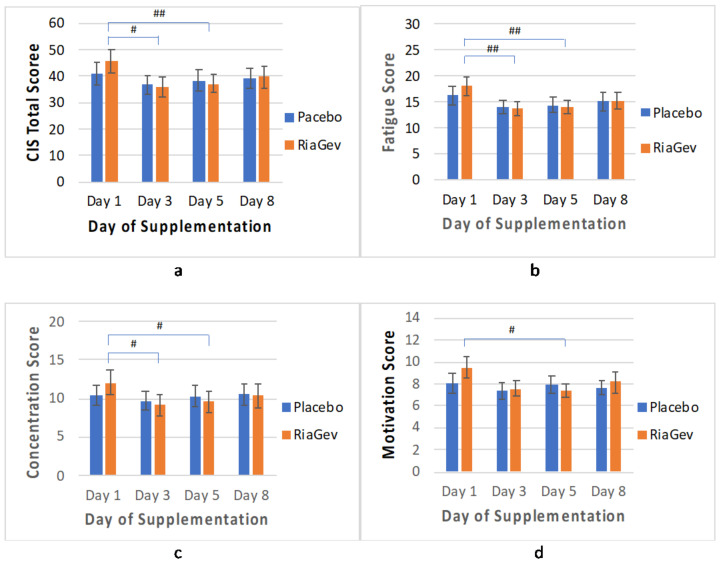
Checklist Individual Strength (CIS) Questionnaire Test for Physical and Mental Fatigue. The CIS total score (**a**) indicates physical and mental tiredness, with four subscales reflecting physical fatigue (**b**), concentration (**c**), motivation (**d**), and physical activity (not shown). Both RiaGev and placebo improved physical and mental fatigue scores during the study. However, the RiaGev group had consistently greater improvements than the Placebo. Additionally, the RiaGev group improvements reached statistically significant levels. Significant changes are marked by # (*p* < 0.05) and ## (*p* < 0.01).

The Checklist Individual Strength (CIS) questionnaire contains a standard set of 20 questions with subscales on physical fatigue, mental concentration, motivation, and physical activities [31]. Both RiaGev and placebo groups showed improvements in CIS scores during the test except in the physical activities subscale (Figure 7). However, improvements in the RiaGev group ware consistently greater than in the placebo group. At Days 3, 5, and 8, the total CIS scores were improved by 21.5% (*p* = 0.04) vs. 10.4% (*p* = 0.07), 18.3% (*p* = 0.014) vs. 6.2% (*p* = 0.049), and 12.7% (*p* = 0.15) vs. 4.1% (*p* = 0.361) in the RiaGev vs. the Placebo group, respectively.

For the subscales, physical fatigue showed the biggest improvement over baseline and biggest difference between the groups. At Days 3, 5, and 8, the physical fatigue improved by 24.3% (*p* = 0.003) vs. 13.6% (*p* = 0.041), 21.2% (*p* = 0.009) vs. 11.6% (*p* = 0.08), 15.1% (*p* = 0.132) vs. 7.4% (*p* = 0.17) in the RiaGev vs. Placebo groups, respectively. Concentration in the RiaGev group also improved significantly by 22.9% (*p* = 0.014), 19.8% (*p* = 0.012) and 14.3% (*p* = 0.118) at Days 3, 5, and 8, respectively, while the improvement in the Placebo group was less significant on any of those days. The same trend was true for motivation, where the RiaGev group improved by 20.4% (*p* = 0.13), 22.2% (*p* = 0.015), and 14% (*p* = 0.163) at Days 3, 5, and 8, respectively, while none of the improvements in the Placebo group reached statistical significance. Among the subscales tested, the physical activity scale did not improve in either RiaGev or Placebo group during the trial periods (data now shown). This is expected because the 7-day supplement period is not long enough to see behavioral changes in terms of daily active level.

### 3.4. Safety Outcomes

#### 3.4.1. Hematology, Clinical Chemistry, Vital Signs, and Anthropometric Measurements

No clinically relevant changes in physical measurements, vital signs, hematology, kidney markers, or electrolytes were observed from pre- to post-supplementation in participants enrolled in this study. All participants were deemed healthy by the PI after both treatment periods.

#### 3.4.2. Incidence of Adverse Events (AEs)

A total of 12 post-emergent AEs were reported by nine participants in this study. Of these, nine minor AEs were reported by seven participants while taking RiaGev and three AEs by two participants while taking the placebo. The reported AEs include decreased appetite, gastrointestinal discomforts, lightheadedness, and weakness, of which only weakness and decreased appetite were considered to be possibly related to the product by the principal investigator.

## 4. Discussion

RiaGev is a proprietary composition combining nicotinamide (NAM, a form of vitamin B3) and D-ribose. It is primarily formulated to efficiently increase the NAD^+^ metabolome via the NAD^+^ salvage pathway. Phosphoribosyl-pyrophosphate (PRPP), an active form of D-ribose, is the co-substrate during NAM salvage, converting NAM to NMN and then to NAD^+^. Each of those two steps requires a molecule of PRPP in the reaction. In individuals with thalassemia minor, their PRPP synthetase activities are below normal and the NAD^+^ levels are significantly decreased in their red blood cells [33]. On the other hand, when human PRPP synthetases 1 and 2 were introduced into *E. coli* under the control of a strong promotor, the maximum amount of NMN was generated when D-ribose was supplied in the cell culture media [34]. Therefore, we predicted that formulating D-ribose with NAM will enhance NAD^+^ production as well as possibly reduce side effects of NAM. More generally, PRPP is a high energy phosphate carrier that drives the production of high energy phosphate molecules, including ATP and ADP. Therefore, we expect an increase in the energy status of the body after RiaGev supplementation. Additionally, D-ribose stimulates NADPH production via the pentose phosphate pathway (PPP), which supplies reducing power in the body [35]. The reducing power is especially important to counterbalance the oxidative nature of NAM and to alleviate the oxidative stress that commonly exists in middle-aged adults [36].

This randomized, triple-blinded, placebo-controlled, cross-over pilot clinical trial demonstrated that supplementation with RiaGev effectively increases concentrations of the NAD^+^ metabolome, especially NADP^+^ levels, in blood (Figure 2). High energy phosphates, including ATP and ADP, and circulating antioxidants, including GSH, were also enhanced (Figure 4 and Figure 5). These results are in line with the predictions of RiaGev formulation. The results from this study also confirm our results in animal studies, showing that RiaGev is more efficient than NAM alone for NAD^+^ metabolome enhancement (to be published).

Along with NAD^+^ and NADP^+^, their reduced forms, NADH and NADPH were also enhanced by RieGev supplementation in animals (to be published). However, we only observed a trending increase of NADPH in this human trial (NADH not measured). Additionally, the NADPH/NADP^+^ and GSH/GSSG ratios are lower than expected, implying oxidation in the human samples even with very careful measures to preserve NADH (Supplementary Materials). During data analysis we noticed that metabolome cofactors pool measurements, including NADPH + NADP^+^ and GSH + GSSG, are better indicators than their individual cofactor measurements. Therefore, we report these cofactor group measurements in this publication.

The discovery that RiaGev primarily enhances NADP^+^ instead of NAD^+^ (27% vs. 10% increase) is unprecedented in NAD^+^ boosting precursor studies. NADP^+^ is a complementary redox coenzyme, which is derived from NAD^+^ or NADPH. An additional ATP is required for its generation by NAD^+^/NADH kinase [37]. Therefore, the higher NADP^+^ yield in the RiaGev group is another indication of its higher energy status.

The pilot clinical trial indicates that the RiaGev group had significantly improved post-prandial glucose tolerance without excessive insulin secretion (Figure 3). NAD^+^ metabolic precursors, including NA, NAM, NMN, and NR, have been widely studied and found to be active for blood glucose control in animal tests [38]. NMN is the only metabolite that demonstrated the benefit of improving blood glucose in human clinical trials so far [39,40]. It was suggested that NAMPT, rather than NAD^+^ metabolome, plays a key regulatory role in insulin secretion and blood glucose clearance [40]. RiaGev derived NAD^+^ production is also catalyzed by NAMPT. It is possible that NAMPT may play a role in RiaGev, improving glucose tolerance. On the other hand, D-ribose is known to cause postprandial hypoglycemia [41]. This acute activity, along with the possible NAMPT mediated regulation, may explain the significant improvement in post-prandial glucose in the pilot trial.

The average baseline blood glucose of the RiaGev group is slightly higher than that of the Placebo group. This baseline imbalance between the groups is also reflected in their hemoglobin A1c during screening (Table 1, 5.50% vs. 5.24% for RiaGev vs. Placebo groups, respectively). Statistical analyses indicate that the imbalances are not significant (*p* > 0.1) and are highly unlikely to disturb the conclusion of this trial. A recent publication [40] on an NMN clinical trial also involved two subject groups whose baseline HbA1c are slightly different (5.7% vs. 5.5% for NMN vs. Placebo groups, respectively). In that case, the group with larger HbA1c was regarded as the prediabetic group. It will be interesting to explore in the future whether the baseline HbA1c imbalance in this RiaGev trial played a similar role as in the NMN study.

Following RiaGev supplementation, the waking salivary cortisol levels in the RiaGev group steadily decreased in contrast to the fluctuating higher levels seen in the placebo group (Figure 6). Waking salivary cortisol is an adrenal stress hormone, which stimulates catabolism that releases glucose into blood stream. When ATP and NAD^+^ are elevated along with glutathione, the sympathetic nervous activity is reduced, which results in less cortisol secretion. Reduction of post-prandial glucose by RiaGev supplementation also means metabolic stress release, which alleviates the body from cortisol secretion. Additionally, NAM may inhibit cortisol action directly. Scott et al. indicated that nicotinamide may be capable of both inhibiting the enzyme responsible for the synthesis of cortisone and reducing its biological activity [42].

With elevated energy and reduced stress, the RiaGev treated group is also presented as having less fatigue, improved concentration, and improved motivation, as reported in the CIS questionnaire (Figure 7). Significant improvements of up to greater than 24% were observed in the CIS scores within the RiaGev group, whereas the improvements in the placebo group did not reach significant level in any category. Like a previous analysis [43], a positively correlation was found between total CIS score and fasting blood glucose (r = 0.952). Meanwhile a negatively correlation was noticeable between total CIS score with NAD+ concentration (r = −0.849). The overall improvement in CIS scores and blood glucose levels during the study observed in both groups is most likely due to the use of dietary and sleep diaries during the clinical trial. We believe that this overall non-significant improvement and short duration of RiaGev supplementation are the main reasons for the non-significant difference between the RiaGev and Placebo groups in the pilot study. A longer study will be needed to address this issue definitively.

It is worth noting that MeNAM and NAAD(P) were not detected in the blood samples with RiaGev supplementation in this study, as they were below the detection limit of the NMR instrument (see the Supplementary materials). MeNAM and NAADP(P) are abundantly common by-products in NR and NAM supplementations [39,44,45], which might contribute to NAM side effects. Combining D-ribose with NAM in RiaGev might reduce MeNAM and NAAD(P) formation, as suggested by their non-detectable levels in this study. NAM phosphorylation, to generate NMN, is a competing reaction to NAM methylation and NAM deamination, which generates MeNAM and NAAD(P), respectively. Thus, it will be of great interest to explore the role of D-ribose that may play in MeNAM and NAAD(P) formation.

RiaGev was found to be safe and well-tolerated in healthy adults between the ages of 35 to 65 years. Only two minor types of adverse events (weakness and decreased appetite) were observed. Notably, there was no skin flushing-related adverse events reported; a common side effect of NAD^+^ precursor supplements. Additionally, no changes were observed in clinical chemistry and hematology.

Although successful, the pilot clinical trial has its limitations. One obvious limitation is its relatively short duration. Since the pilot trial is the first clinical trial for RiaGev, we designed the human pilot study based on our preclinical animal studies. This made the last clinical visit (Day 8) too long, such that it pushed the sampling into the afternoon, much later than the sampling time on Days 1, 3, and 5. This sampling time difference is believed to be the main reason that measurements on Day 8 did not completely follow the trend of Day 3 and Day 5, resulting in lower than expected measurements in the NAD^+^ metabolome as well as from the CIS questionnaire. Previous studies indicate the NAD^+^ metabolome is highly regulated by circadian rhythm [4]. A portion of the cellular NAD^+^ pool breaks down into NAM and ADP-ribose during the night and they are resynthesized into NAD^+^ by the following morning. Therefore, the cellular NAD^+^ level oscillates daily, and the afternoon hours typically show lower levels of the NAD^+^ metabolome than in the morning.

The limitations of this pilot clinical trial were successfully addressed by another independent human study (result to be published separately), where more subjects (27 vs. 18) and a longer supplementation (4 weeks vs. 1 week) were employed along with a newly developed home-based NAD^+^ testing method [46]. The result from this extended study indicates that blood NAD^+^ levels increase almost linearly with supplementation in the first two weeks to about 125% of baseline, well beyond the one-week 110.4% in the pilot trial. The blood NAD^+^ level continues increasing, although at a reduced rate, to week 4 to reach 127% of the baseline. The NAD^+^ level on Day 3 and Day 5 of the pilot trial are consistent with their corresponding levels in the extended study, respectively, while the Day 8 result is well below its corresponding value. This supports the notion that lower than expected NAD^+^ measurements on Day 8 in the pilot trial was due to inconsistent timing of measurement. Therefore, this data should not be considered to be a correct Day 8 value. On the other hand, it means that results and conclusions from pilot trial are conservative because they were mostly derived from Day 3 and Day 5 measurements only and the correct (should-be stronger) Day 8 result was not considered. Moreover, this extended study also suggests that the conclusions drawn from this pilot study are true and relevant to general consumers for everyday use.

Due to the favorable safety profile of RiaGev and its strong improvement in the NAD^+^ metabolome and blood glucose levels, future work should expand the population to subjects at risk of reduced NAD^+^ levels, such as the elderly, those with impaired glucose tolerance, and those suffering from the intricate aspects of metabolic syndrome including prediabetes and diabetes. More generally, people suffering from oxidative stress would benefit from RiaGev because D-ribose would provide reductive protection [35]. Compared to NR and NMN, NAM is inexpensive and has a long history of safety [12]. The combination of D-ribose and NAM in RiaGev improves NAM metabolism, which may improve human performance and provide a new approach to enhance the NAD^+^ metabolome.

## 5. Conclusions

This randomized, triple-blind, placebo-controlled, crossover pilot study assessed the efficacy and safety of a combination of NAM and D-ribose (RiaGev) in healthy adults. Supplementation with RiaGev effectively increased concentrations of the NAD^+^ metabolome, especially NADP^+^ levels, in circulating blood. It also enhanced the high energy phosphate and glutathione levels in the blood. The RiaGev group had significantly improved post-prandial glucose levels without excess insulin secretion, suggesting improved glucose tolerance. The circulating antioxidants, including GSH, were also enhanced with RiaGev. The waking cortisol was also consistently lower in the RiaGev group than in the placebo group. In addition, a CIS questionnaire assessment suggests that RiaGev may reduce physical fatigue, improve concentration, improve motivation, as well as overall well-being of the subjects, although further studies with a longer duration of supplementation are needed to confirm their significance. Taken together, all these parameters are consistent with one another, suggesting that RiaGev is effective in enhancing the NAD^+^ metabolome, thus bringing about related benefits in middle-aged adults.

RiaGev was found to be safe and well-tolerated in healthy adults. Only two minor adverse events (weakness and decreased appetite) were observed. Notably, there was no skin flushing-related adverse events reported, a common side effect of NAD^+^ precursor supplements. Additionally, no changes were observed in clinical chemistry and hematology. This favorable safety profile of RiaGev suggests that the combination of D-ribose with NAM may help to expand the usage of this B3 vitamin for broad application and human benefit.

## 6. Patents

A patent based on this this study has been applied under the Patent Cooperation Treaty (PCT) US22/70209.

## Figures and Tables

**Table 1:** Subjects Demographics and Grouping. The subjects were randomized and divided into two matching groups based on their demographic and physical information, such as age, sex, weight, and height. The BMI, heart rate, and hemoglobin A1c of each group were also not statistically different. The clinical trial of each group proceeded with either Placebo-RiaGev sequence or RiaGev-Placebo sequence in cross-over design (Figure 1).

Parameter	Placebo—RiaGev Mean ± SD (*n*) Median (Min–Max)	RiaGev—Placebo Mean ± SD (*n*) Median (Min–Max)	Between Groups *p*-Value
Age (years)	53.3 ± 7.3 (9)	50.1 ± 8.6 (9)	0.405
	53 (39 to 64)	53 (38 to 62)	
Gender			1.000
Female [*n* (%)]	6 (66.7%)	5 (55.6%)	
Male [*n* (%)]	3 (33.3%)	4 (44.4%)	
Weight (kg)	79.1 ± 20.0 (9)	75.2 ± 14.6 (9)	0.642
	68.8 (57.8 to 114.4)	70.3 (59.4 to 102.2)	
Height (cm)	172.7 ± 14.2 (9)	175.2 ± 8.1 (9)	0.645
	166.0 (157.0 to 200.0)	172.0 (165.0 to 189.0)	
BMI (kg/m^2^)	26.1 ± 2.8 (9)	24.3 ± 3.0 (9)	0.193
	27.2 (21.0 to 28.9)	23.7 (20.3 to 28.8)	
Heart Rate (bpm)	66.3 ± 9.1 (9)	64.1 ± 19.6 (9)	0.243
	63 (55 to 80)	60 (49 to 112)	
Hemoglobin A1c (%)	5.24 ± 0.37	5.50 ± 0.25	0.108
	5.2 (4.8 to 6.0)	5.4 (5.2 to 6.0)	

## Data Availability

The data of this study are also published in ClinicalTrails.gov under identifier NCT04483011.

## Supplementary Materials

The following supporting information can be downloaded at: https://www.mdpi.com/article/10.3390/nu14112219/s1. The results of this pilot trial is published in ClinicalTrials.gov registered under identifier NCT04483011. (https://clinicaltrials.gov/ct2/show/results/NCT04483011, accessed on 21 April 2022).
