# Supplementary material for: A Combination of Nicotinamide and D-Ribose (RiaGev) Is Safe and Effective to Increase NAD+ Metabolome in Healthy Middle-Aged Adults: A Randomized, Triple-Blind, Placebo-Controlled, Cross-Over Pilot Clinical Trial
Source: Nutrients. 2022 May 26;14(11):2219. doi: 10.3390/nu14112219 (PMC9183138; doi:10.3390/nu14112219)
Supplement: Supplementary file 1 [file nutrients-14-02219-s001.zip › nutrients-1701095-supplementary.pdf]

Supplementary Materials for Nutrents-1701095

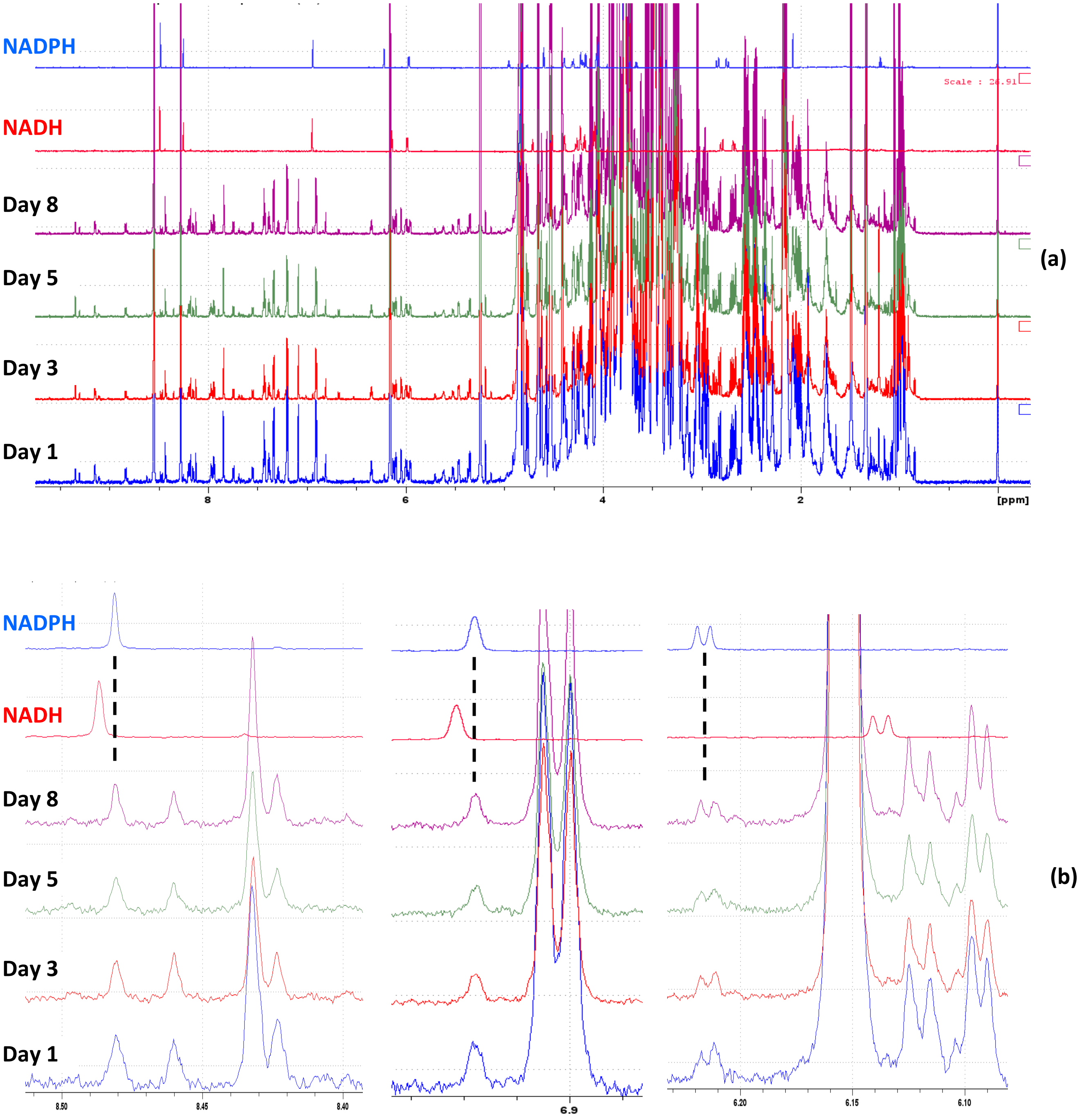

Figure S1: Preservation of NADPH Destroyed NADH. (a) 800 MHz ^{1}H NMR spectra of four blood samples from a subject obtained at days 1, 3, 5 and 8 after administering RiaGev (NAM + D-ribose). Standard NADH and NADPH spectra obtained under identical conditions as for blood are shown for comparison with blood spectra; (b) Expanded regions of the blood spectra from (a) highlighting the assignment of NADPH peak. It is clear from the spectra that NADH is not detected in the blood by NMR because the method used to preserve NADPH in blood storage and transportation destroyed NADH.

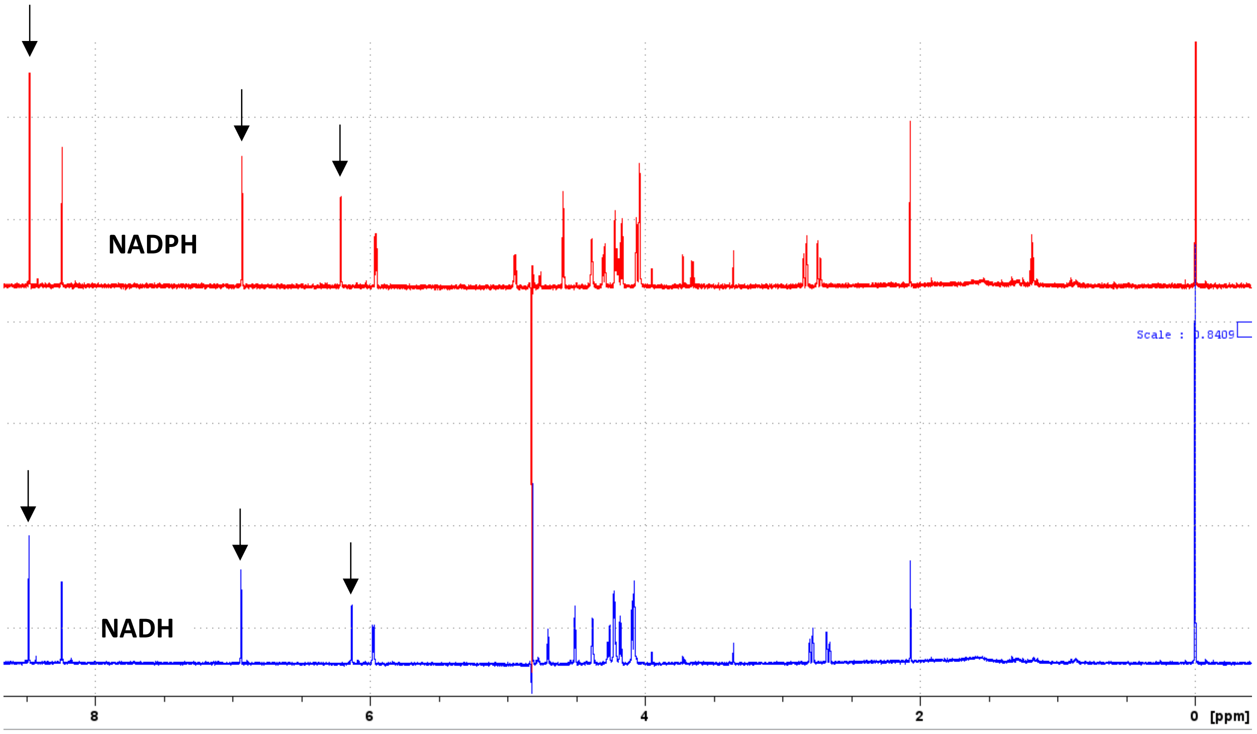

Figure S2: 800 MHz ^{1}H NMR spectra of standard NADH and NADPH compounds obtained under conditions as used for blood samples. Peaks that are clearly identifiable in blood spectra are pointed with arrows.

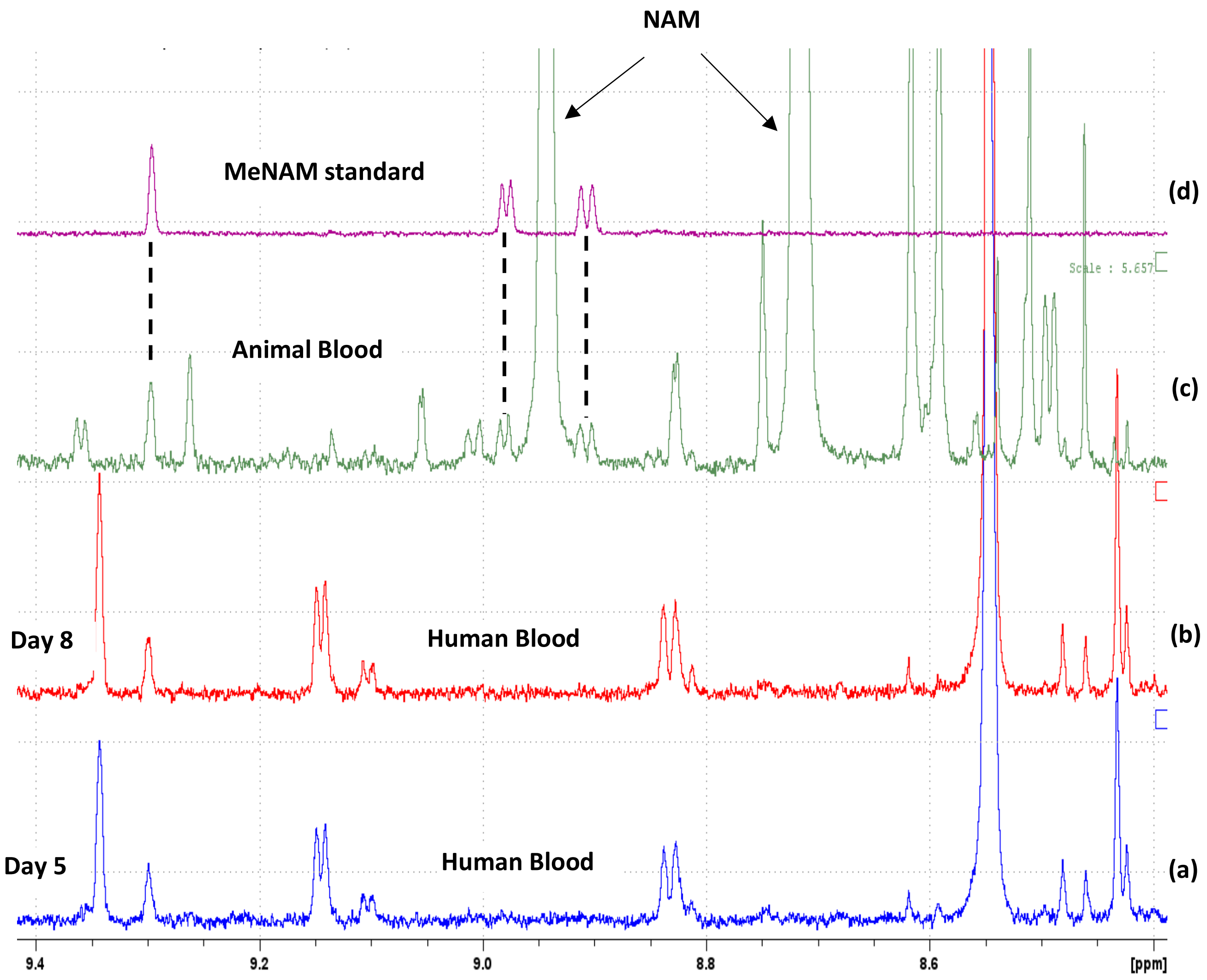

Figure S3: N-Methyl-Nicotinamide (MeNAM) Not Detectable in the Pilot Trial. Parts of 800 MHz ^{1}H NMR spectra of (a, b) human blood, (c) animal blood and (d) standard N-methylnicotinamide (MeNAM) highlighting the presence of MeNAM peaks in the animal blood and not in human blood. Both human and animal were administered with RiaGev (NAM + D-ribose); however, its doses were 6x - 45x higher for the animal than human on the per kilogram bodyweight basis as indicated by the strong NAM peaks in the animal blood (unpublished work). Day 5 and Day 8 indicate the number of days of continuous RiaGev administration in human trial. NAM was not detected by NMR in human blood even on Day 8, which is the longest RiaGev supplementation in the human pilot trial (19RNHB).
